# Hygroma of the Subcutaneous Bursæ on the Ischiatic Promontories in Cattle

**Published:** 1897-12

**Authors:** 


HYGROMA (CHRONIC HYDROPS) OF THE SUBCUTANEOUS BURSÆ ON THE ISCHIATIC PROMONTORIES IN CATTLE.—If we are to judge the occurrence of this disease according to the statements which are found in the literature upon it, then we must assume that hygroma of the kind mentioned does not occur. Neither

Müller, Hoffmann, nor Fröhner make mention of the trouble in their books on general surgery. The following contribution may prove that hygroma on the ischial promontory of cattle occurs comparatively often, and, perhaps on account of its harmlessness, has up till now received little attention on the part of owners and veterinarians.

As official veterinarian of the State Cattle Insurance Establishment in Baden, hygroma in cattle came to my notice, while I had never met with the same elsewhere in my private practice. The fact that three cases of the same occurred during the course of a year shows that chronic hydrops of the sacs in the mucous membrane of certain parts of the bodies of cattle can occur not infrequently, and this is further confirmed by the etiology. In all three cases I have found this swelling only on one side and always on the ischiatic promontory of that side which was turned toward a post or wall. The animals leaned mostly against these, and the prerequisites of the disease, in the shape of contusions, bruises, etc., were thus caused. The narrow stalls, which are usually fitted up very scantily, and leave little room for the animal to move around, easily explain the fact that the occurrence of hygroma is more frequent than is generally known.

Two of these patients leaned against a post situated directly behind them, and the third had the habit, while eating, of resting against a stone which projected from the wall. This observation agrees with the fact that young dogs which are not yet full-grown, and which are kept in narrow kennels, are usually affected with such ischiatic swellings. The hygroma in all cases consisted of a swelling varying in size from the fist to that of a child's head. It was in most cases compact, movable, and not very painful, and the skin could easily be moved over it. The vertical diameter was double that of the horizontal. When an incision was made in the tumor a thin serous fluid, varying in color from a reddish-brown to a reddish-yellow, flowed out, and when it was probed with the finger it showed a spongy cellular formation. In two cases I found, also, one larger and several smaller whitish-yellow ovoidal bodies (corpora oryzoïdea), which were connected with the base by thin stems, and which could be easily separated with the finger. These little bodies reached the size of a pigeon-egg, and generally showed fibrinous strands, forming cavities which resembled the crystal groups of aragonite, well known to mineralogists. The whole growth was confined to the subcutaneous tissue, and only in one case, in which the tumor was as large as a child's head, was the sinewy part of the muscles found in a semi-membranous mangled condition.

Healing took place very rapidly. After an incision from 6–8 cm. long has been made, the corpora oryzoïdea should be removed with the finger and the tumor squeezed out like a sponge, a wad of oakum soaked in tincture of iodine is then inserted, and should be renewed daily. In the course of a week the hygroma disappeared and the wound healed up. External application of iodine preparations on the tumor, without opening, are too tedious and cause the owner much trouble.—Römer, *Idem.*

